# Distinct roles of COMPASS subunits to *Drosophila* heart development

**DOI:** 10.1242/bio.061736

**Published:** 2024-10-17

**Authors:** Jun-yi Zhu, Joyce van de Leemput, Zhe Han

**Affiliations:** ^1^ Center for Precision Disease Modeling, Department of Medicine, University of Maryland School of Medicine, 670 West Baltimore Street, Baltimore, MD 21201, USA; ^2^ Division of Endocrinology, Diabetes, and Nutrition, Department of Medicine, University of Maryland School of Medicine, 670 West Baltimore Street, Baltimore, MD 21201, USA

**Keywords:** Heart development, Histone modification, *Drosophila*, COMPASS complex, Common components, Unique components

## Abstract

The multiprotein complexes known as the complex of proteins associated with Set1 (COMPASS) play a crucial role in the methylation of histone 3 lysine 4 (H3K4). In *Drosophila*, the COMPASS series complexes comprise core subunits Set1, Trx, and Trr, which share several common subunits such as ash2, Dpy30-L1, Rbbp5, and wds, alongside their unique subunits: Wdr82 for Set1/COMPASS, Mnn1 for Trx/COMPASS-like, and Ptip for Trr/COMPASS-like. Our research has shown that flies deficient in any of these common or unique subunits exhibited high lethality at eclosion (the emergence of adult flies from their pupal cases) and significantly shortened lifespans of the few adults that do emerge. Silencing these common or unique subunits led to severe heart morphological and functional defects. Moreover, specifically silencing the unique subunits of the COMPASS series complexes, *Wdr82*, *Mnn1*, and *Ptip*, in the heart results in decreased levels of H3K4 monomethylation and dimethylation, consistent with effects observed from silencing the core subunits Set1, Trx, and Trr. These findings underscore the critical roles of each subunit of the COMPASS series complexes in regulating histone methylation during heart development and provide valuable insights into their potential involvement in congenital heart diseases, thereby informing ongoing research in heart disease.

## Teaser

**Summary:** This research conducts a systematic research to identify the roles of both common and unique COMPASS subunits in *Drosophila* heart development.

## INTRODUCTION

The complex of proteins associated with Set1 (COMPASS) family members counteract the polycomb group (PcG) protein-mediated transcriptional repression. COMPASS competes for target binding sites and replaces PcG repressive marks, thereby, in general, promoting an active state of chromatin (Miller et al., 2001; Schwartz et al., 2010). As such, COMPASS plays significant roles in transcriptional regulation, development, and disease pathogenesis (Shilatifard, 2012). The COMPASS series complex is conserved from yeast (*Saccharomyces cerevisiae*) through human (Mohan et al., 2011; Shilatifard, 2012). In mammals, the lysine methyltransferase (KMT/mixed lineage leukemia, MLL) family genes KMT2A, KMT2B, KMT2C, KMT2D, SETD1A, and SETD1B encode core subunits of the COMPASS series complexes (Qian and Zhou, 2006; Shilatifard, 2012). Notably, *de novo* mutations in KMT2A have been identified in patients with congenital heart disease (CHD) (Ji et al., 2020; Jinxiu et al., 2020). Furthermore, 18 mutations in KMT2C have also been reported in patients with CHD (Borland et al., 2019). Consistently, a mouse model with a deleted SET domain in KMT2C exhibited abnormal heart development and ventricular septal defects (Ang et al., 2016). KMT2D, another crucial component, is known to regulate cardiac gene expression during heart development primarily via H3K4 dimethylation (Ang et al., 2016). Loss of KMT2D has led to significant heart defects in both mice and zebrafish models (Ang et al., 2016; Schwenty-Lara et al., 2019; Serrano et al., 2019; Van Laarhoven et al., 2015). Additionally, mutations in KMT2D have been linked to Kabuki syndrome, a congenital disorder characterized by multiple malformations, including distinct craniofacial features, with 70% of these patients presenting with CHD (Bokinni, 2012; Digilio et al., 2017). In the previous study, we identified the distinct roles for the COMPASS core subunits Set1 (mammalian SETD1A and SETD1B), Trx (mammalian KMT2A and KMT2B), and Trr (mammalian KMT2C and KMT2D) in *Drosophila* heart development (Huang et al., 2022; Zhu et al., 2023). These studies demonstrate the importance of the core subunits of the COMPASS series complex in heart development and their association with CHD.

**Fig. 1.**
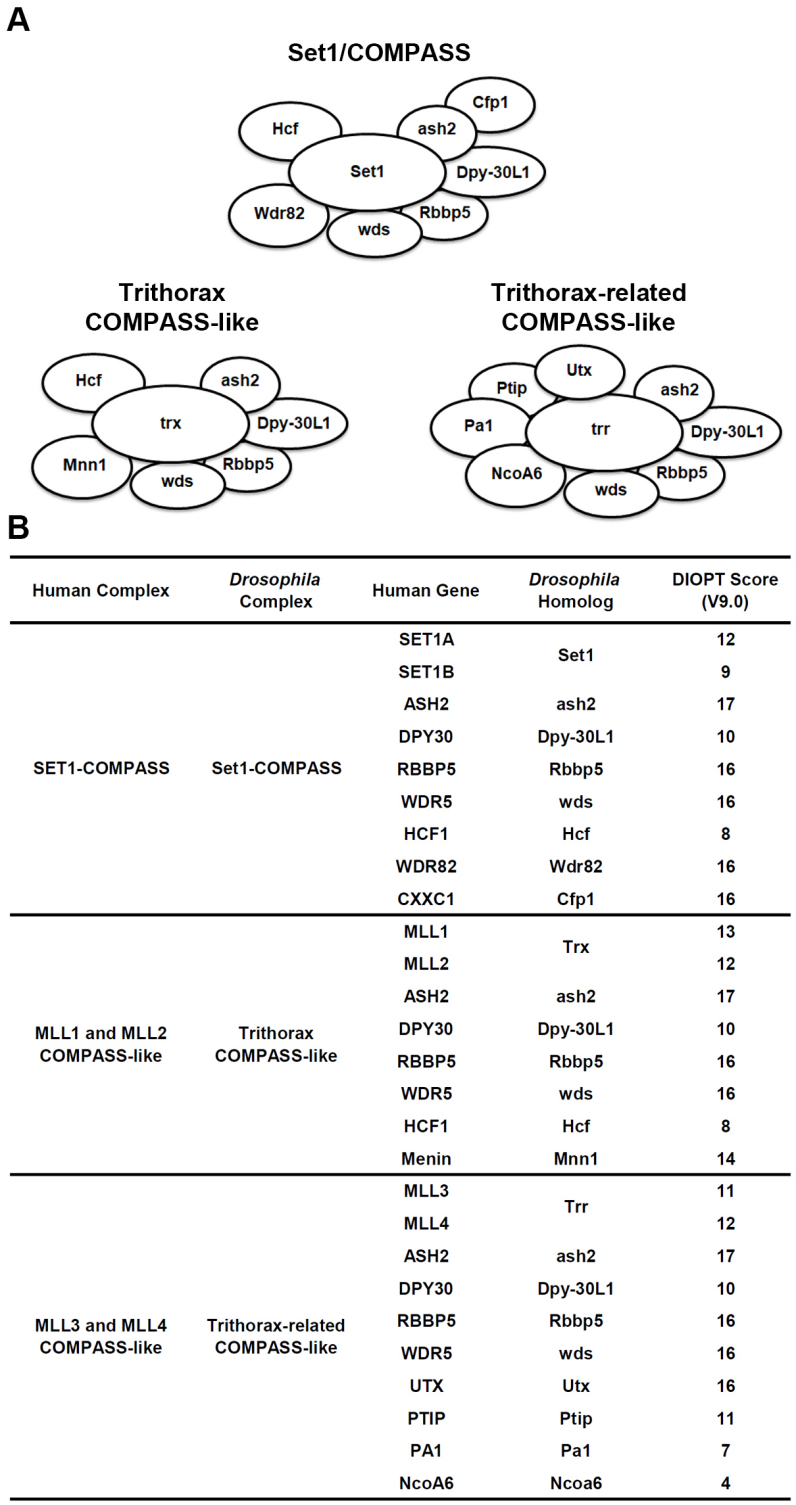
***Drosophila* COMPASS complexes and their subunits.** (A) Schematic representation of three different *Drosophila* COMPASS complexes: Set1/COMPASS, Trithorax (Trx) COMPASS-like, and Trithorax-related (Trr) COMPASS-like complex. Ash2, Absent, small, or homeotic discs 2; Cfp1, CXXC finger protein 1; Dpy-30L1, Dpy-30-like 1; Hcf, Host cell factor; Mnn1, Menin 1; Ncoa6, Nuclear receptor coactivator 6; Pa1, PTIP associated 1; Ptip, PAX transcription activation domain interacting protein; Rbbp5, Retinoblastoma binding protein 5; Set1, SET domain containing 1; Utx, Utx histone demethylase; Wdr82, WD repeat domain 82; Wds, Will die slowly. (B) Human and *Drosophila* COMPASS complexes and their subunits. DIOPT, DRSC integrative ortholog prediction tool (version 9.0; max score fly-human 19).

Each of the COMPASS series complexes shares several common subunits: Ash2 (mammalian ASH2L), Dpy-30L1 (mammalian DPY30), Rbbp5 (mammalian RBBP5), and Wds (mammalian WDR5), collectively known as the WRAD (or WARD) module. These subunits are essential for the assembly of the COMPASS complexes. In addition to these shared components, each COMPASS complex contains subunits specific to one or a few COMPASS families, which are likely involved in chromatin recruitment. These include (1) host cell factor (Hcf; mammalian HCF1), a subunit for Set1 COMPASS and Trx COMPASS-like. (2) WD repeat domain 82 (Wdr82; mammalian WDR82) and CXXC finger protein 1 (Cfp1; mammalian CXXC1), exclusive to Set1/COMPASS. (3) Menin 1 (Mnn1; mammalian Menin), specific to Trx/COMPASS-like. (4) PAX transcription activation domain interacting protein (Ptip; mammalian PTIP), Utx histone demethylase (Utx; mammalian UTX), PTIP associated 1 (Pa1; mammalian PA1), and nuclear receptor coactivator 6 (Ncoa6; mammalian NcoA6), which are distinct to Trr/COMPASS-like (Fig. 1A). These varying subunits confer distinct functions to the multi-subunit COMPASS complexes. Like the methyltransferase core units, these subunits are highly conserved, as indicated by the DRSC integrative ortholog prediction tool (DIOPT) (Fig. 1B). This conservation underscores their critical roles across species and emphasizes the functional importance of each component in the broader context of chromatin modification and gene regulation. While several subunits such as Wdr5, HCF1, and UTX have been linked to CHD (Homsy et al., 2015; Jin et al., 2017; Priest et al., 2016; Zaidi et al., 2013; Zhu et al., 2017b), the roles of most other subunits in heart development and their association with heart diseases remain largely unknown. This gap in knowledge highlights the need for further research to understand the contributions of these components in cardiac physiology and pathology.

To further elucidate the roles of the common and unique subunits of COMPASS series complexes in heart development, we utilized *Drosophila*, a well-established model system for studying cardiac development and disease (Zhu et al., 2017b). Silencing the common subunits of the COMPASS series complexes, *ash2*, *Dpy30-L1*, *Rbbp5*, and *wds*, led to severe morphological and functional defects in the heart. Furthermore, silencing the unique subunits of the COMPASS series complexes, *Wdr82*, *Mnn1*, and *Ptip*, not only resulted in similar heart defects but also significantly reduced H3K4 monomethylation (H3K4me1) and dimethylation (H3K4me2). These effects were consistent with those observed from silencing the core subunits *Set1*, *Trx*, and *Trr*. Taken together, our findings highlight the critical roles of each subunit within the COMPASS series complexes in regulating histone methylation during heart development. These insights contribute to our understanding of their potential involvement in CHD and are invaluable for guiding ongoing research into cardiovascular disease.

## RESULTS

### Silencing COMPASS common components *ash2*, *Dpy-30L1*, *Rbbp5*, or *wds* impacted *Drosophila* survival

**Fig. 2.**
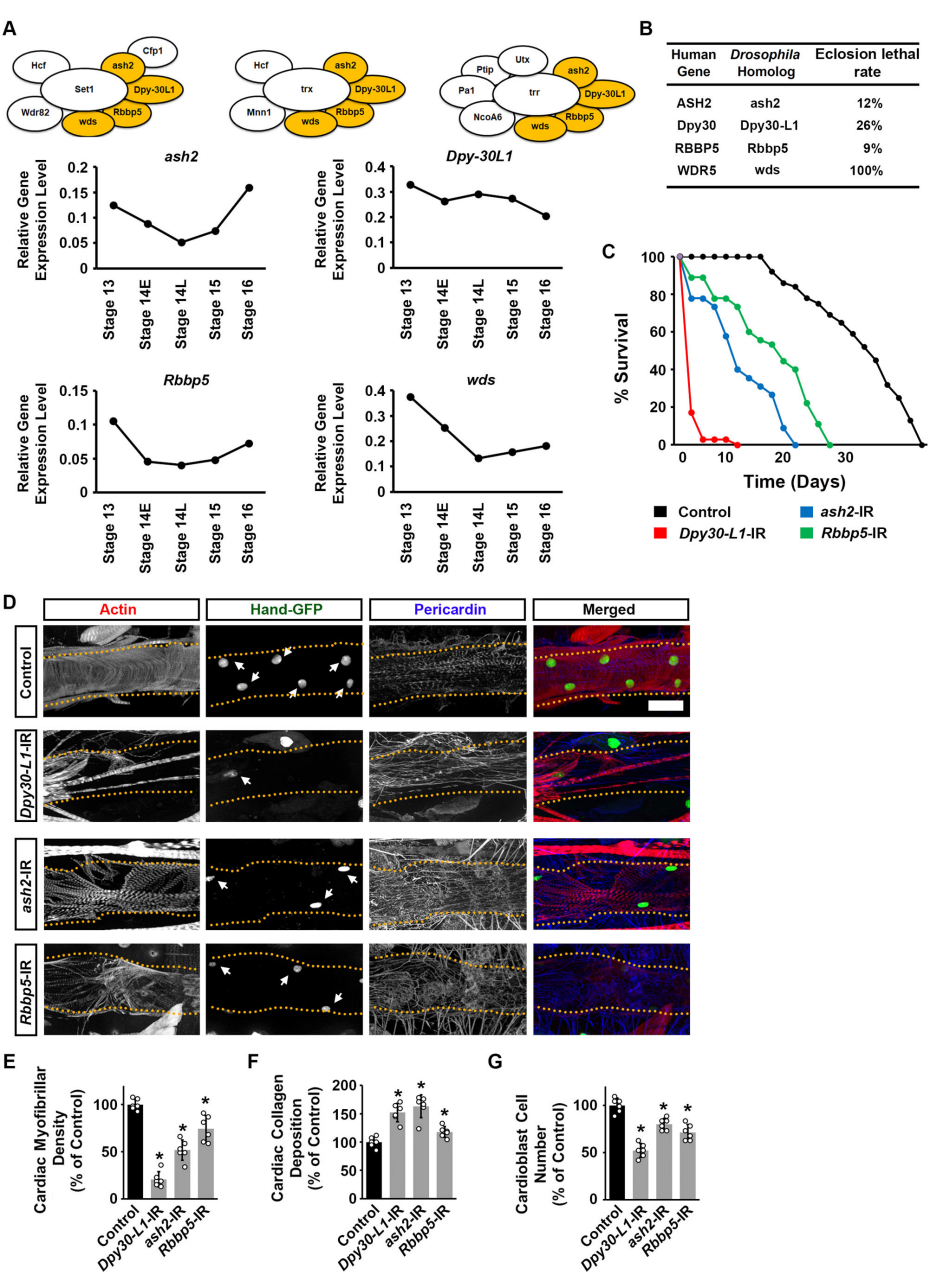
***Drosophila* survival and heart structure following heart-specific silencing of common COMPASS subunits.** (A) Above, the three *Drosophila* COMPASS complexes: Set1/COMPASS, Trithorax (Trx) COMPASS-like, and Trithorax-related (Trr) COMPASS-like complex with the three shared subunits highlighted in yellow: Ash2, Absent, small, or homeotic discs 2; Dpy-30L1, Dpy-30-like 1; and Wds, Will die slowly. Below, is the relative gene expression level for *ash2*, *Dpy30-L1*, and *wds* in *Drosophila* heart (cardiogenic progenitors) at embryonic stages 13, 14-E (early), 14-L (late), 15, and 16. None of the changes reached statistical significance. (B) Eclosion lethality induced by 4X*Hand*-Gal4-driven expression of UAS-RNAi transgenes targeting *ash2*, *Dpy30-L1*, or *wds*. By crossing with a CyO (curly wing) balancer, emerging adults with curly wings (CyO) have no transgene expression, whereas those with straight wings express 4X*Hand*-Gal4>RNAi. The eclosion lethal rate (percent) is calculated as [((curly − straight) / curly) × 100]. All lines also expressed 4X*Hand*-Gal4. *n*=400+ flies (female and male) per genotype. (C) Survival curves for adult flies expressing *ash2*, or *Dpy30-L1* RNAi (-IR) transgenes (4X*Hand*-Gal4) in heart cells; and *Hand*-GFP. Control, *Hand*-GFP;*4XHand*-Gal4^+/−^. *n*=100 male flies (20 flies/vial) per genotype. (D) Representative images for adult (4-day-old females) cardiac structure following the expression of UAS-RNAi transgenes targeting *ash2* or *Dpy30-L1* (4X*Hand*-Gal4). Cardiac actin myofibers were visualized by phalloidin staining (red). *Hand*-GFP expression (green; nuclear) labels cardiomyocytes (heart cells). Pericardin was detected by immunofluorescence (blue). Dotted lines delineate the outline of the heart tube. Arrows point to heart cardiomyocytes. Control, *Hand*-GFP;4X*Hand*-Gal4^+/−^. Scale bar: 40 µm. (E) Quantitation of adult heart cardiac myofibrillar density relative to control (see image in D) [mean±s.d.; *n*=6 flies (4-day-old females) per genotype; Kruskal–Wallis H-test followed by a Dunn's test; statistical significance: *P<0.05]. (F) Quantitation of adult heart cardiac collagen (Pericardin) deposition relative to control (see images in D) [mean±s.d.; *n*=6 flies (4-day-old females) per genotype; Kruskal–Wallis H-test followed by a Dunn's test; statistical significance: *P<0.05]. (G) Quantitation of adult heart cardiomyocyte numbers relative to control (see images in D) [mean±s.d.; *n*=6 flies (4-day-old females) per genotype; Kruskal–Wallis H-test followed by a Dunn's test; statistical significance: *, *P*<0.05].

First, we investigated the expression levels of the genes that encode the shared COMPASS subunits: *absent*, *small*, or *homeotic discs 2* (*ash2*), *Dpy-30-like 1* (*Dpy-30L1*), *Retinoblastoma binding protein 5* (*Rbbp5*) and *will die slowly* (*wds*). We used single-cell RNAseq data obtained from cells collected during critical embryonic stages of *Drosophila* heart development; from the migration of bilateral rows of cardiac progenitors (stage 13 onward) to the formation of a closed heart tube (stage 16) (Huang et al., 2023). The expression of *ash2*, *Dpy-30L1*, *Rbbp5*, and *wds* was steady in the cardiac progenitor cells throughout these embryonic development stages (Fig. 2A).

**Fig. 3.**
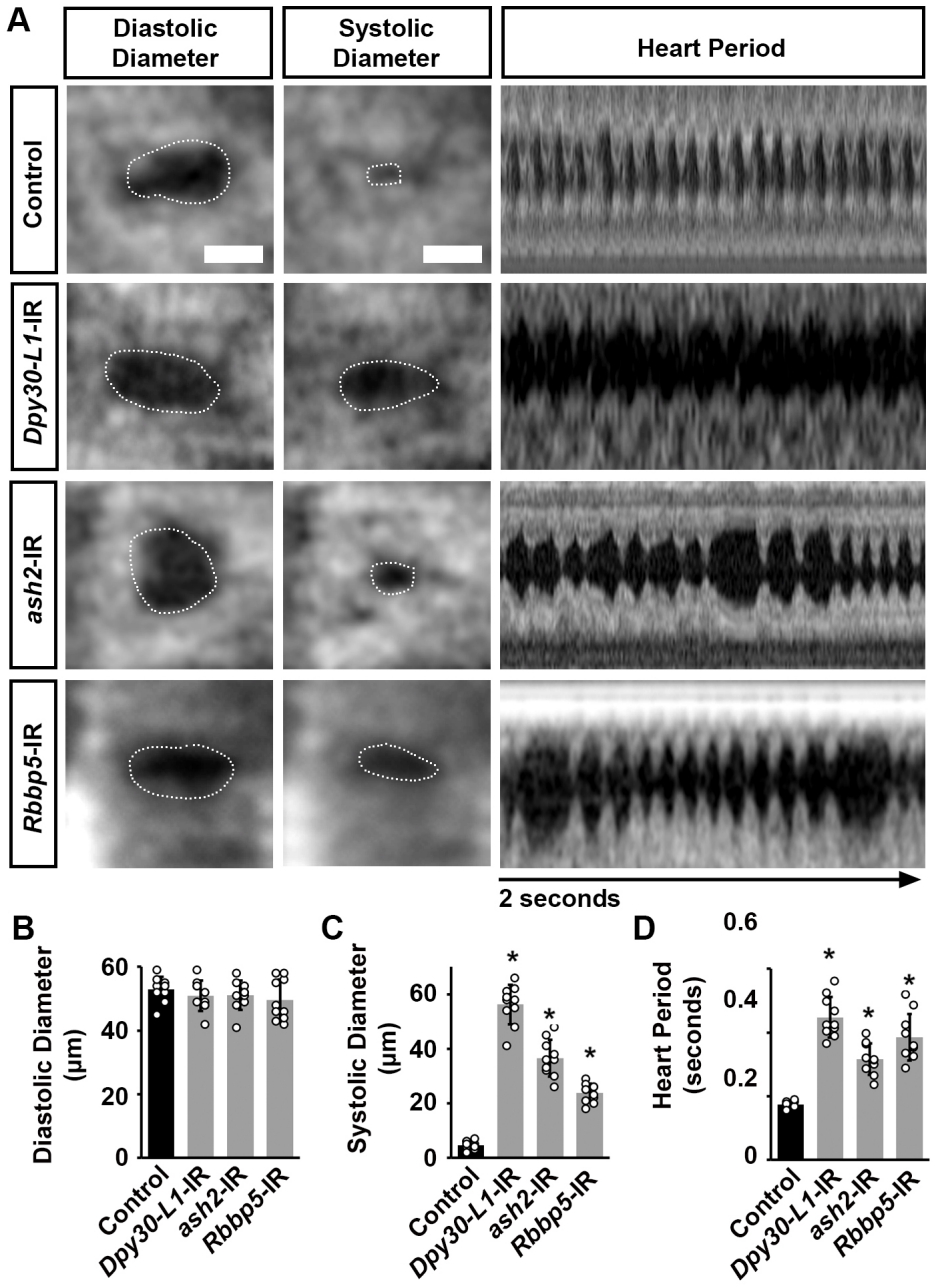
**Cardiac function in flies following heart-specific silencing of common COMPASS subunits.** (A) Images from *Drosophila* (4-day-old females) heartbeat videos obtained by OCT. Representative images for heart function shown for flies that express UAS-RNAi transgenes targeting absent, small, or homeotic discs 2 (*ash2*) or Dpy-30-like 1 (*Dpy-30L1*) (driven by 4X*Hand*-Gal4); each RNAi (-IR) line also expressed *Hand*-GFP. Control, *Hand*-GFP;4X*Hand*-Gal4^+/−^. The dotted line outlines the circumference of the heart tube. Scale bars: 20 μm. (B) Quantitation of adult heart diastolic diameter (see images in A) [mean±s.d.; *n*=10 flies (4-day-old females) per genotype; Kruskal–Wallis H-test followed by a Dunn's test; statistical significance was defined as *P*<0.05]. (C) Quantitation of adult heart systolic diameter (see images in A) [mean±s.d.; *n*=10 flies (4-day-old females) per genotype; Kruskal–Wallis H-test followed by a Dunn's test; statistical significance: *P<0.05]. (D) Quantitation of heart period (see images in A) [mean±s.d.; *n*=10 flies (4-day-old females) per genotype; Kruskal–Wallis H-test followed by a Dunn's test; statistical significance: *, *P*<0.05].

Then, we used the *Drosophila* UAS-Gal4 system to knock down the expression of each of the shared COMPASS complex components specifically in the heart. This was achieved by combining the heart-specific Gal4 driver, 4X*Hand*-Gal4 (Zhu et al., 2017b), with UAS-*ash2*-RNAi, UAS-*Dpy-30L1*-RNAi, UAS-*Rbbp5*-RNAi, or UAS-*wds*-RNAi constructs. Two independent RNAi fly lines were tested for each (*ash2*, *Dpy-30L1*, *Rbbp5*, and *wds*). Since these showed similar results, representative data for one line each have been displayed in the figures (data for lines *ash2*-IR ID 64942, *Dpy-30L1*-IR ID 41946, *Rbbp5*-IR ID 42819, and *wds*-IR ID 32952 are shown in Figs 2 and 3). Silencing *wds*, resulted in complete (100%) lethality at eclosion, i.e. emergence of adult fly from the pupal case (Fig. 2B). Silencing *ash2*, *Dpy30-L1* or *Rbbp5* led to reduced eclosion rates (Fig. 2B), and the adult flies that did emerge showed dramatically shorted lifespans compared to control flies (4X*Hand*-Gal4^+/−^) (Fig. 2C). These data demonstrate the importance of the shared COMPASS subunits, Ash2, Dpy-30L1, Rbbp5, and Wds, in the heart for *Drosophila* development.

### Silencing COMPASS complex common components induced cardiac structural and functional defects in adult *Drosophila*

Silencing *ash2*, *Dpy-30L1*, or *Rbbp5* in the fly heart led to early lethality and a reduced lifespan. Thus next, we wanted to examine the effects on the heart. Since no adult flies emerged for the 4X*Hand*>*wds*-RNAi line, these flies were not investigated as adults. Previously, we observed that heart-specific silencing of *wds* resulted in abnormal heart morphology in late larvae, characterized by reduced muscle fiber density and significant over-deposition of Pericardin, a marker of *Drosophila* cardiac tissue integrity (Zhu et al., 2017b). The overabundance of Pericardin indicates a pathophysiological condition of fibrosis and can be used as an indicator of cardiac injury (Hughes et al., 2020; Ren et al., 2023; Vaughan et al., 2018).

Silencing either *ash2*, *Dpy-30L1*, or *Rbbp5* in the heart resulted in disorganized cardiac actin filaments (visualized by phalloidin stain; Fig. 2D) and decreased cardiac muscle fiber density (Fig. 2D and E). Moreover, the accumulation of Pericardin, was significant in *ash2*, *Dpy-30L1*, and *Rbbp5* silenced flies (Fig. 2D and F). Finally, cardiomyocyte numbers (visualized by green fluorescent protein drive by a *Hand* gene regulatory element) were significantly reduced when *ash2*, *Dpy-30L1*, or *Rbbp5* were silenced in the heart (Fig. 2D and G).

To assess any cardiac functional defects induced by silencing *ash2*, *Dpy-30L1*, or *Rbbp5*, we applied optical coherence tomography (OCT). OCT is the fly equivalent of an electrocardiogram (ECG) in humans. The OCT's orthogonal view of the *Drosophila* heart enables real-time measurements of the heart tube diameter and heart period (Fig. 3A). Silencing *ash2*, *Dpy-30L1*, or *Rbbp5* in the fly heart was not associated with changes in the diastolic diameter (Fig. 3B); however, the systolic diameter was significantly increased for all of them (Fig. 3C). In addition, the heart period in *ash2*, *Dpy-30L1*, or *Rbb5* silenced flies was significantly increased compared to control flies (4X*Hand*-Gal4^+/−^) (Fig. 3D).

Altogether, these findings show that the common COMPASS components are necessary for normal heart structure and function. The more severe heart phenotypes observed in flies deficient in common COMPASS components highlight their critical importance across all three COMPASS complex series. These findings suggest that the common subunits may be involved in both H3K4me1 and H3K4me2 methylation.

### Silencing unique COMPASS subunits *Wdr82*, *Mnn1*, or *Ptip* impacted *Drosophila* survival

**Fig. 4.**
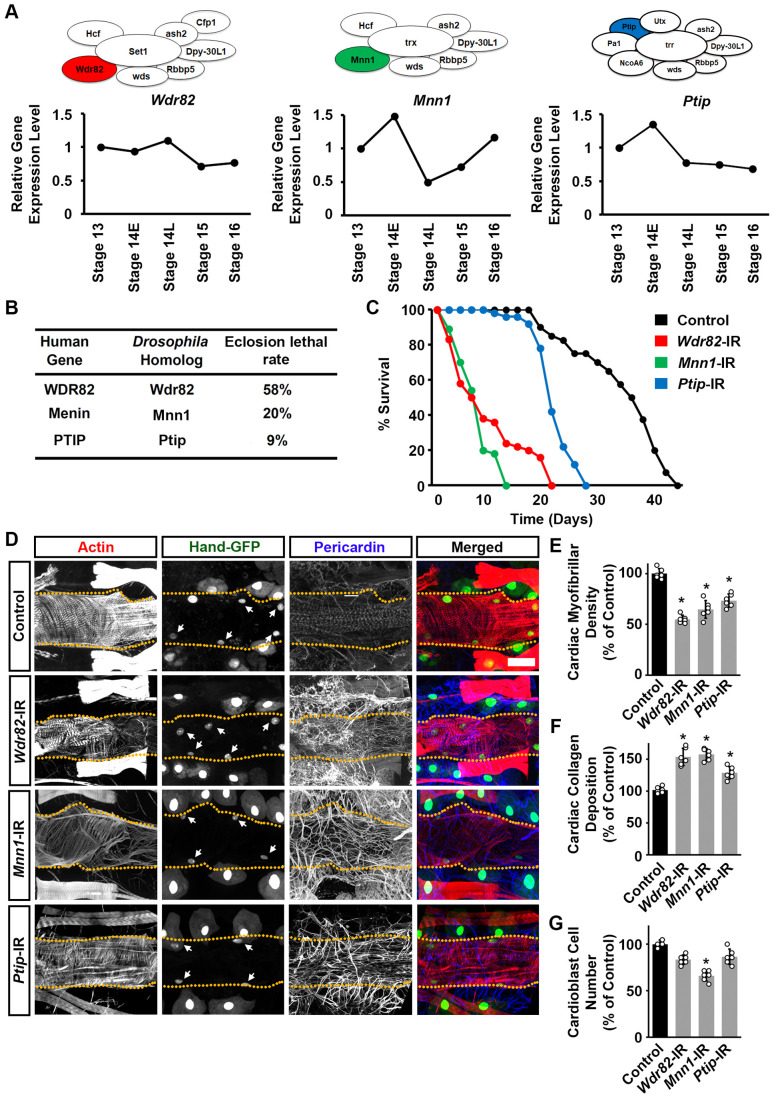
***Drosophila* survival and heart structure following heart-specific silencing of unique COMPASS subunits.** (A) Above, the three *Drosophila* COMPASS complexes: Set1/COMPASS, Trithorax (Trx) COMPASS-like, and Trithorax-related (Trr) COMPASS-like complex with a subunit unique to each complex highlighted: WD repeat domain 82 (Wdr82), Menin 1 (Mnn1), and PAX transcription activation domain interacting protein (Ptip), respectively. Below, is the relative gene expression level for *Wdr82*, *Mnn1*, and *Ptip* in *Drosophila* heart (cardiogenic progenitors) at embryonic stages 13, 14-E (early), 14-L (late), 15, and 16. None of the changes reached statistical significance. (B) Eclosion lethality induced by 4X*Hand*-Gal4-driven expression of UAS-RNAi transgenes targeting *Wdr82*, *Mnn1,* or *Ptip*. By crossing with a CyO (curly wing) balancer, emerging adults with CyO have no transgene expression, whereas those with straight wings express 4X*Hand*-Gal4>RNAi. The eclosion lethal rate (percent) is calculated as [((curly − straight) / curly) × 100]. All lines also expressed *Hand*-Gal4. *n*=400+ flies (female and male) per genotype. (C) Survival curves for adult flies expressing *Wdr82*, *Mnn1*, or *Ptip* RNAi (-IR) transgenes (4X*Hand*-Gal4) in heart cells; and, *Hand*-GFP. Control, *Hand*-GFP;4X*Hand*-Gal4^+/−^. *n*=100 male flies (20 flies/vial) per genotype. (D) Representative images for adult (4-day-old females) cardiac structure following the expression of UAS-RNAi transgenes targeting *Wdr82*, *Mnn1*, or *Ptip* (4X*Hand*-Gal4). Cardiac actin myofibers were visualized by phalloidin staining (red). *Hand*-GFP expression (green; nuclear) labels cardiomyocytes (heart cells). Pericardin was detected by immunofluorescence (blue). Dotted lines delineate the outline of the heart tube. Arrows point to heart cardiomyocytes. Control, *Hand*-GFP;4X*Hand*-Gal4^+/−^. Scale bar: 40 µm. (E) Quantitation of adult heart cardiac myofibrillar density relative to control (see images in D) [mean±s.d.; *n*=6 flies (4-day-old females) per genotype; Kruskal–Wallis H-test followed by a Dunn's test; statistical significance: *, *P*<0.05]. (F) Quantitation of adult heart cardiac collagen (Pericardin) deposition relative to control (see images in D) [mean±s.d.; *n*=6 flies (4-day-old females) per genotype; Kruskal–Wallis H-test followed by a Dunn's test; statistical significance: *, *P*<0.05]. (G) Quantitation of adult heart cardiomyocyte numbers relative to control (see images in D) (mean±s.d.; *n*=6 flies (4-day-old females) per genotype; Kruskal–Wallis H-test followed by a Dunn's test; statistical significance: *P<0.05).

Next, we investigated subunits that are unique to the COMPASS complexes: WD repeat domain 82 (Wdr82; Set1/COMPASS); Menin 1 (Mnn1; Trx/COMPASS-like); and PAX transcription activation domain interacting protein (Ptip; Trr/COMPASS-like). We started with their gene expression levels in the early-developing fly embryonic heart using the single-cell RNAseq data (Huang et al., 2023). The expressions of *Wdr82*, *Mnn1*, and *Ptip* in the cardiac progenitor cells were steady throughout the embryonic heart development stages; however, while not quite reaching significance, Trr/COMPASS-like unique subunit *Ptip* showed a drop in expression from stage 14E (Fig. 4A). The gene expression patterns of the unique subunits in the three COMPASS series complexes are similar to those of their respective core subunits, *Set1*, *trx*, and *trr* (Fig. 4A) (Zhu et al., 2023).

**Fig. 5.**
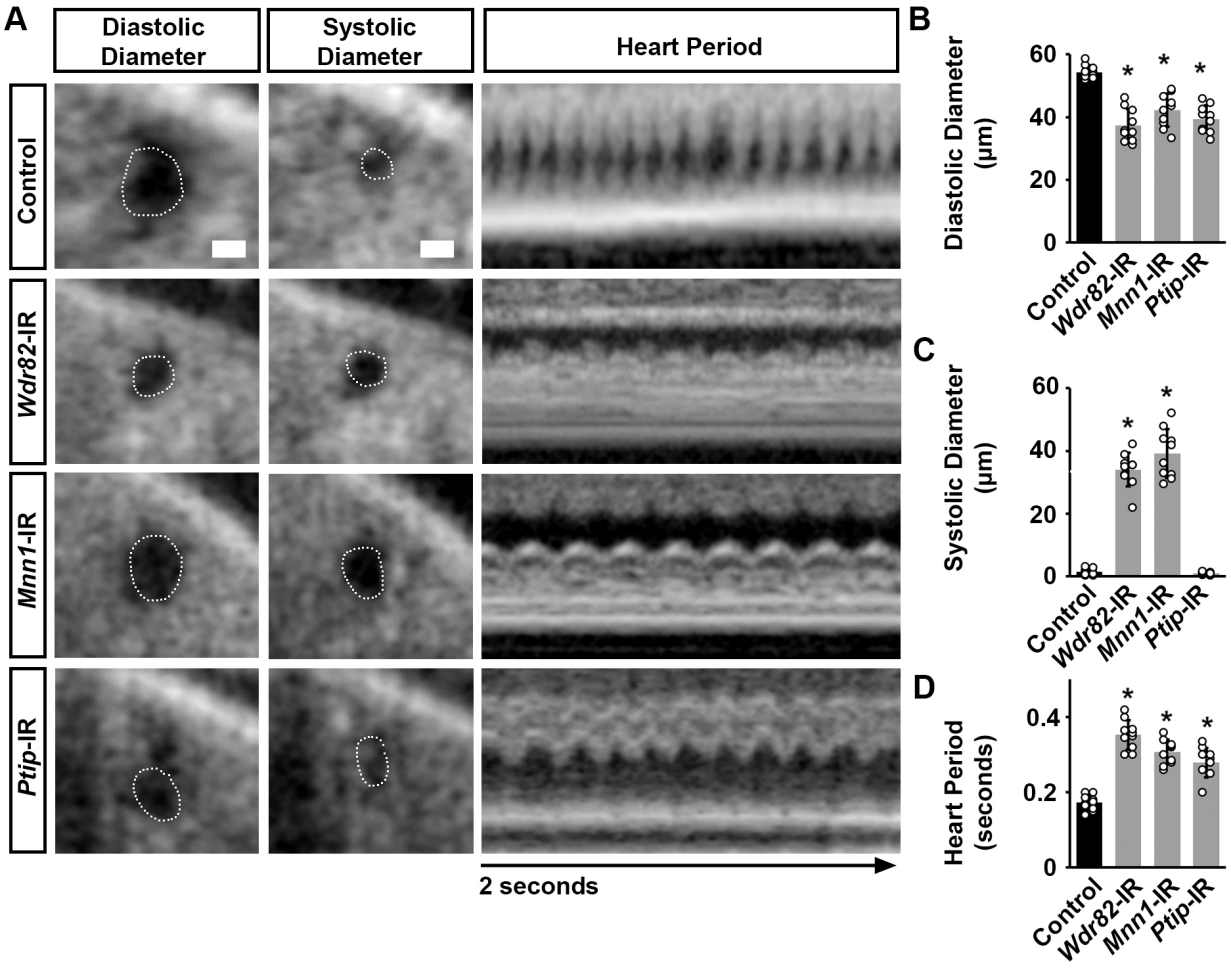
**Cardiac function in flies following heart-specific silencing of unique COMPASS subunits.** (A) Images from *Drosophila* (4-day-old females) heartbeat videos obtained by OCT. Representative images for heart function are shown for flies that express UAS-RNAi transgenes targeting WD repeat domain 82 (*Wdr82*), Menin 1 (*Mnn1*), or PAX transcription activation domain interacting protein (*Ptip*) (driven by 4X*Hand*-Gal4); each RNAi (-IR) line also expressed *Hand*-GFP. Control, *Hand*-GFP;4X*Hand*-Gal4^+/−^. The dotted line outlines the circumference of the heart tube. Scale bars: 20 μm. (B) Quantitation of adult heart diastolic diameter (see images in A) [mean±s.d.; *n*=10 flies (4-day-old females) per genotype; Kruskal–Wallis H-test followed by a Dunn's test; statistical significance: *, *P*<0.05]. (C) Quantitation of adult heart systolic diameter (see images in A) [mean±s.d.; *n*=10 flies (4-day-old females) per genotype; Kruskal–Wallis H-test followed by a Dunn's test; statistical significance: *, *P*<0.05]. (D) Quantitation of heart period (see images in A) [mean±s.d.; *n*=10 flies (4-day-old females) per genotype; Kruskal–Wallis H-test followed by a Dunn's test; statistical significance: *, *P*<0.05].

**Fig. 6.**
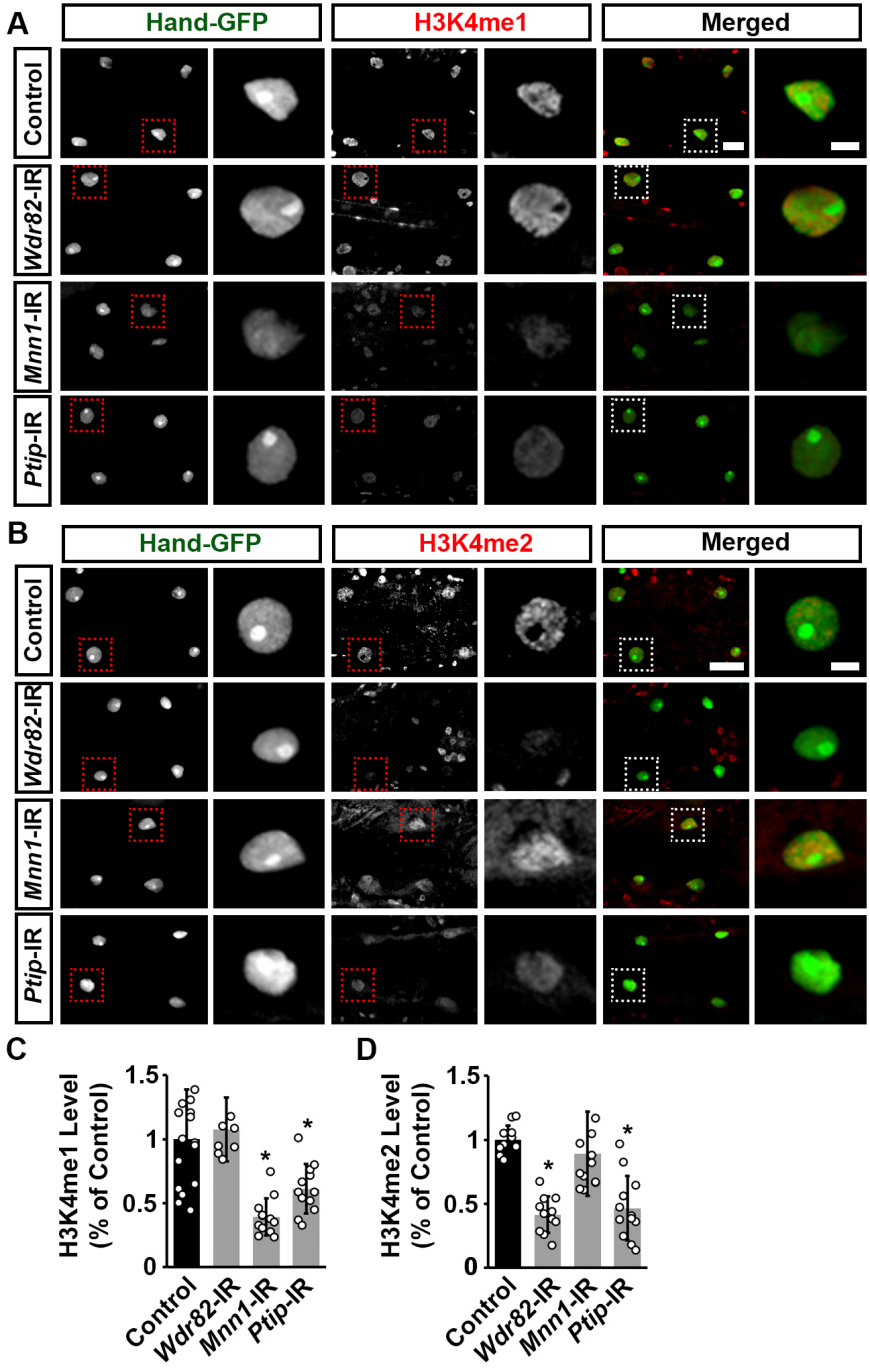
**H3K4 methylation levels in *Drosophila* cardiomyocytes following heart-specific silencing of unique COMPASS subunits.** (A,B) Representative images for adult heart H3K4 methylation: (A) monomethylation (H3K4me1); and (B) dimethylation (H3K4me2). Flies (4-day-old females) expressed UAS-RNAi transgenes targeting WD repeat domain 82 (*Wdr82*), Menin 1 (*Mnn1*), or PAX transcription activation domain interacting protein (*Ptip*) (driven by 4X*Hand*-Gal4); each RNAi (-IR) line also expressed *Hand*-GFP. Control, *Hand*-GFP;4X*Hand*-Gal4^+/−^. The dotted box indicates the magnified area, to show the cardiomyocyte nucleus. Scale bars: 20 µm. Scale bars (magnification) 5 µm. (C) Quantitation of adult heart cardiac H3K4me1 level relative to the level in control flies (*Hand*-GFP;*4XHand*-Gal4^+/−^) (see images in A). [Mean±s.d.; *n*=24 nephrocytes from six flies (4-day-old females), four cardiomyocytes each] per genotype; Kruskal–Wallis H-test followed by a Dunn's test; statistical significance: *P<0.05. (D) Quantitation of adult heart cardiac H3K4me2 level relative to the level in control flies (*Hand*-GFP;*4XHand*-Gal4^+/−^) (see images in B). Mean±s.d.; *n*=24 nephrocytes from six flies (4-day-old females), four cardiomyocytes each] per genotype; Kruskal–Wallis H-test followed by a Dunn's test; statistical significance: *P<0.05.

Next, we again used the heart-specific Gal4 driver, 4X*Hand*-Gal4 (Zhu et al., 2017b) to knock down the expression of each of the unique COMPASS complex components in the heart. Two independent RNAi lines were tested for each (*Wdr82*, *Mnn1*, and *Ptip*). These showed similar results, therefore representative data for one line each have been displayed in the figures (data for lines *dr82*-IR ID 32926, *Mnn1*-IR ID 35150, and *Ptip*-IR ID 35269 are shown in Figs 4, 5, and 6). Silencing *Wdr82* or *Mnn1* in the fly heart causes high mortality at eclosion (Fig. 4B) as well as markedly shortened lifespans (Fig. 4C). Cardiac-specific silencing of *Ptip* also increased lethality at eclosion but to a lesser extent (Fig. 4B), while lifespan showed a normal progression until after 20 days at which point survival rapidly dropped compared to control flies (4X*Hand*-Gal4^+/−^) (Fig. 4C). These results demonstrate the importance of the unique COMPASS subunits, Wdr82, Mnn1, and Ptip, in the heart for *Drosophila* development.

### Silencing unique COMPASS subunits induced cardiac structural and functional defects in adult *Drosophila*

A closer look at the heart showed notably disorganized cardiac actin filaments and significantly decreased cardiac muscle fiber density (visualized by phalloidin stain) following knockdown of *Wdr82*, *Mnn1*, or *Ptip* in the fly heart (Fig. 4D and E). Accumulation of Pericardin was significantly increased in *Wdr82*, *Mnn1*, and *Ptip* silenced flies (Fig. 4D and F). In addition, the *Mnn1*-IR flies showed a significantly reduced number of cardiomyocytes (Fig. 4G).

We again used OCT to assess any cardiac functional defects induced by silencing each of the unique COMPASS subunits (Fig. 5A). Silencing *Wdr82*, *Mnn1*, or *Ptip* was associated with a significantly reduced diastolic diameter (Fig. 5B). Silencing *Wdr82* or *Mnn1*, but not *Ptip*, resulted in a significantly increased systolic diameter (Fig. 5C). For all three, the heart period was significantly increased compared to control flies (4X*Hand*-Gal4^+/−^) (Fig. 5D).

Together these findings show that each of the three unique COMPASS complex components, Wdr82, Mnn1, and Ptip, is required for normal heart structure and function.

### The unique COMPASS subunits regulate H3K4 mono- and dimethylation in the *Drosophila* heart

Previously, we showed that the COMPASS complex core units Set1, Trx, and Trr are essential in the epigenetic regulation during *Drosophila* heart development (Zhu et al., 2023). Therefore, we next examined the roles of the unique COMPASS subunits Wdr82, Mnn1, and Ptip in H3K4 methylation in the adult fly heart using immunofluorescence. Since we previously showed that H3K4me3 could not be detected in the adult *Drosophila* heart (Huang et al., 2022; Zhu et al., 2023), we focused on H3K4 mono(me1)- and di(me2)-methylation. In typical control flies, H3K4me1 and H3K4me2 were observed in the nucleus of cardiomyocytes (Fig. 6A and B). Notably, when silenced, each of the unique complex subunits showed a different change in H3K4 methylation in the *Drosophila* cardiomyocyte nuclei: *Wdr82*-RNAi did not change H3K4me1 levels, but significantly reduced H3K4me2; *Mnn1*-RNAi led to significantly reduced H3K4me1, but no changes in H3K4me2 levels; and *Ptip*-RNAi led to significant reductions in both H3K4me1 and H3K4me2 (Fig. 6A-D). These data indicate the significant contributions of each of the unique COMPASS subunits to the regulation of H3K4 methylation by their respective complexes in the fly cardiomyocytes.

## DISCUSSION

COMPASS series complexes are crucial for H3K4 methylation, which is associated with gene expression activity (Shilatifard, 2012). These complexes regulate transcription factor access to chromatin, transcriptional fidelity, and splicing outcomes (Collins et al., 2019). Each COMPASS series complex is highly conserved across species, from flies to mammals (Huang et al., 2022; Mohan et al., 2011; Shilatifard, 2012; Takahashi et al., 2011). In *Drosophila*, each complex features a unique core subunit (Set1, Trx, or Trr) and shares several common subunits such as ash2, Dpy30-L1, Rbbp5, and wds, in addition to unique subunits: Wdr82 for Set1/COMPASS, Mnn1 for Trx/COMPASS-like, and Ptip for Trr/COMPASS-like. Our previous studies have highlighted distinct roles for the core subunits Set1, Trx, and Trr in heart development, mediated through different levels of H3K4 methylation across embryonic stages (Zhu et al., 2023). Set1 and Trr influence several metabolic pathways in early developmental stages, and Set1 is active throughout development, while Trx is pivotal in regulating muscle and heart differentiation during later stages (Zhu et al., 2023). The roles of the common and unique subunits of each COMPASS series complex in heart development remain less understood. In this study, we show that the common subunits ash2, Dpy30-L1, Rbbp5, and wds are essential for *Drosophila* heart development. Previous studies have also demonstrated the necessity of these common subunits for heart development in various animal models. *Ash2L*, the human homolog of *Drosophila ash2*, is broadly expressed in early mouse embryos and works alongside TBX1 to regulate histone methyltransferase activity, crucial for heart development (Stoller et al., 2010). *RBBP5*, the human homolog of *Drosophila Rbbp5*, interacts with the transcription factor *c-Jun*, primarily involved in DNA transcription (Dunn et al., 2002). The loss of *c-Jun* leads to increased *RBBP5* expression, which enhances H3K4me3 deposition on key genes essential for cardiogenesis (Zhong et al., 2023). *WDR5*, the human homolog of *Drosophila wds*, plays a significant role in cell formation and is implicated in CHD (Kulkarni and Khokha, 2018; Kulkarni et al., 2018; Zhu et al., 2017b). Although the human homolog of *Drosophila* Dpy30-L1, DPY30, is suggested to influence human embryonic stem cell fate decisions by modulating H3K4 methyltransferase activity (Bertero et al., 2015), its specific role in heart development remains less defined. This points to a complex interplay of these COMPASS subunits across different species, highlighting their pivotal roles in cardiac developmental processes.

**Fig. 7.**
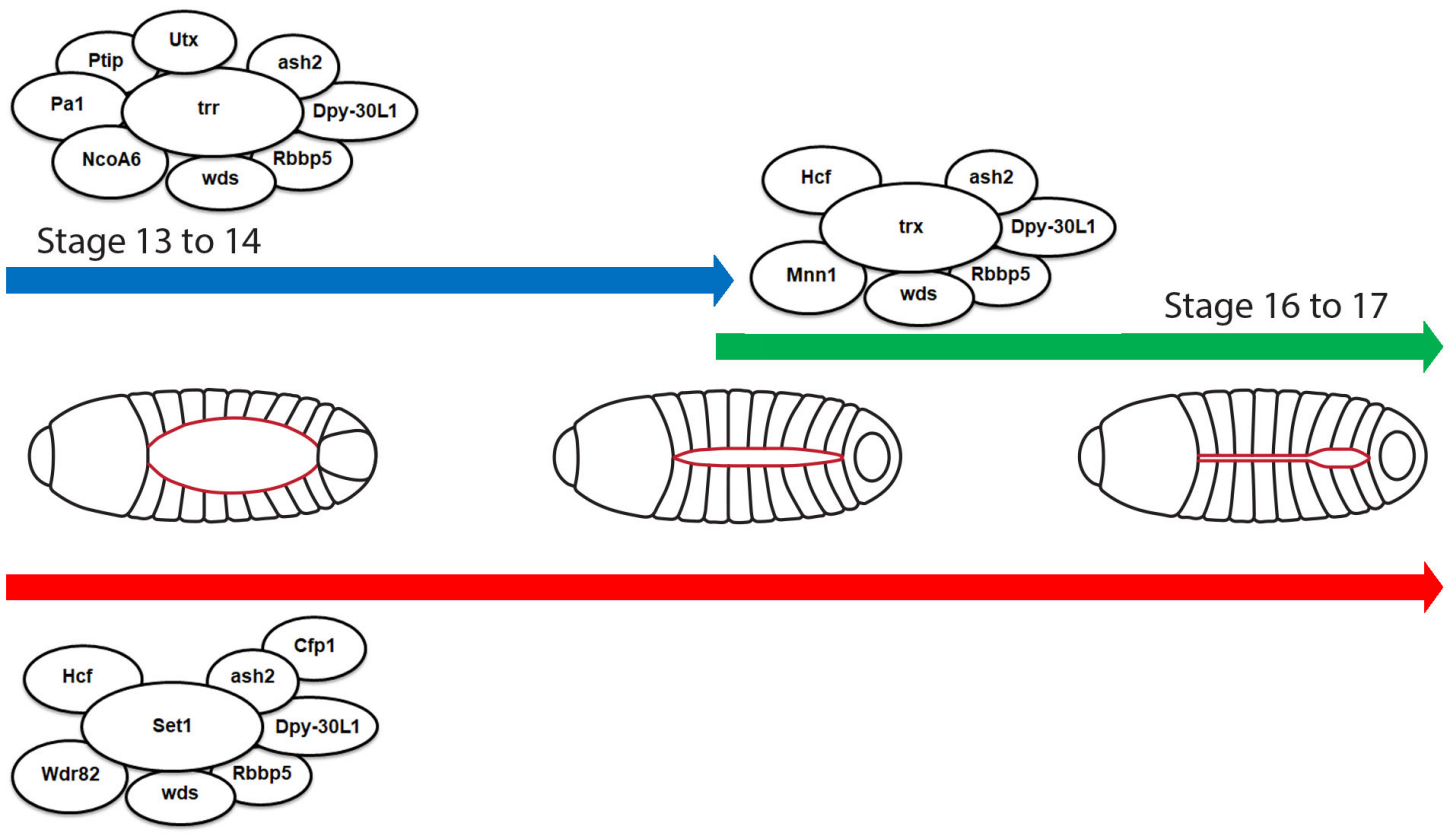
**Model of COMPASS series complexes mediated *Drosophila* heart development.** Graphical representation of the proposed model for the roles of Set1/COMPASS, Trx/COMPASS-like, and Trr/COMPASS-like in mediating fly heart development. COMPASS series complexes comprise core subunits Set1, Trx, and Trr, which share several common subunits such as ash2, Dpy30-L1, Rbbp5, and wds, alongside their unique subunits. The graphical model is divided by the established *Drosophila* developmental stages. The developing heart, represented in red, is shown in fly larvae. At the early stages (stages 13-14), Heart progenitor cells begin their migration. During these stages, Set1/COMPASS and Trr/COMPASS-like are prominently active, initiating early developmental signals in the heart tissue; during later stages (stages 16-17), The heart matures into a closed tube. Set1/COMPASS continues its activity, supporting further development and maturation of heart structures, while Trx/COMPASS-like gradually takes over the role of Trr/COMPASS-like, facilitating the final stages of heart development.

Furthermore, the unique subunits of each COMPASS series complex exhibit gene expression trends similar to their corresponding core subunits. For example, both the core subunit Trr and its unique subunit Ptip from the Trr/COMPASS-like complex display decreasing gene expression from early developmental stages (Huang et al., 2023; Zhu et al., 2023). Similarly, the methylation levels regulated by the core subunits and their respective unique subunits align: Set1 and its unique subunit Wdr82 both regulate H3K4me2, while Trx and its unique subunit Mnn1 regulate H3K4me1. Trr and its unique subunit Ptip influence both H3K4me1 and H3K4me2. Collectively, our data indicate that each COMPASS series complex facilitates distinct methylation states during *Drosophila* heart development (Fig. 7), suggesting a concerted action where each subunit is vital for healthy heart development. This understanding could extend to other animal models, indicating a universal mechanism for heart development regulation through COMPASS complexes.

Advances in genomic analysis techniques have significantly enhanced our ability to identify a broad array of genes that encode products with diverse functions potentially linked to heart-related diseases, such as CHD. Variants in several components of the COMPASS series complexes, such as HCF1, WDR5, and KMT2D, have been discovered in patients with CHD (Homsy et al., 2015; Zaidi et al., 2013). However, the likelihood that a genetic variant identified in a CHD-related gene is causative of the disease is relatively low (Forrest et al., 2022; Sun et al., 2022). Even though modern bioinformatic tools can classify these variants as loss-of-function, gain-of-function, or variants with uncertain effects, validation through *in vivo* animal models is essential. *Drosophila* has proven to be a crucial model for studying heart development and associated diseases due to the remarkable conservation of gene function across species (Ahmad, 2017; Souidi and Jagla, 2021; Taghli-Lamallem et al., 2016; Zhang and Saunders, 2017). In our studies, we have validated that variants in WDR5 identified in CHD patients have a direct association with the disease (Zhu et al., 2017b). Similarly, other variants in the COMPASS series complexes can also be validated using *Drosophila*, providing a robust model to explore the genetic underpinnings of heart development and the mechanisms by which these genetic factors contribute to heart diseases.

Altogether, as demonstrated in previous research, each of the COMPASS series complexes mediates distinct methylation levels and fulfills various roles at different stages of heart development (Zhu et al., 2023). Our findings further reinforce that all core, common, and unique subunits play essential roles in heart development (Fig. 7).

## MATERIALS AND METHODS

### *Drosophila* lines

*Drosophila* stocks were obtained from the Bloomington *Drosophila* Stock Center (BDSC; Indiana University Bloomington, IN, USA) and the Vienna *Drosophila* Resource Center (VDRC; Vienna, Austria). The following lines were used in this study: UAS-*ash2*-RNAi (BDSC ID 35388 and 64942), UAS-*Dpy-30L1*-RNAi (BDSC ID 41946 and VDRC ID 27625), UAS-Rbbp5-RNAi (BDSC ID 42819 and VDRC ID 106139), UAS-*wds*-RNAi (BDSC ID 32952 and 60399), UAS-*Wdr82*-RNAi (BDSC ID 32926 and VDRC ID 25246), UAS-*Mnn1*-RNAi (BDSC ID 31220 and 35150), and UAS-*Ptip*-RNAi (BDSC ID 31741 and 35269). Control *w^1118^* (BDSC ID 3605) flies were used in the crosses. The 4X*Hand*-Gal4/Cyo (Zhu et al., 2017b) driver was generated in our lab and used to express RNAi-based silencing (-IR) constructs in the heart. The *Hand*-GFP fly line was generated previously (Han and Olson, 2005).

### Lethality at eclosion

Eclosion lethality is evidenced by the percentage of flies expressing an RNAi-based silencing construct (straight wings) that fail to emerge as adults, relative to siblings that do not express the silencing construct (curly wings). The result has been presented as the eclosion lethal rate (percentage), calculated as [((curly - straight)/curly)×100]. At least 400 flies (female and male) were counted per genotype.

### Adult *Drosophila* survival assay

*Drosophila* larvae were kept at 25°C to induce UAS-transgene expression. Adult male flies were subsequently maintained in vials at 25°C, with each vial containing 20 flies. Mortality was monitored every 48 h. One hundred flies were assayed per genotype.

### Immunochemistry

Adult flies (4-day-old females) were dissected and fixed for 10 min in 4% paraformaldehyde in phosphate-buffered saline (1X PBS). Primary antibodies were incubated overnight at 4°C in 2% bovine serum albumin (BSA; Sigma, St. Louis, MO, USA) with 0.1% Triton-X (Sigma, St. Louis, MO, USA) in 1X PBS. Following washes, secondary antibodies were incubated for 2 h at room temperature in 2% BSA (Sigma, St. Louis, MO, USA) with 0.1% Triton-X (Sigma, St. Louis, MO, USA) in 1X PBS. Alexa Fluor 488 phalloidin (Thermo Fisher, Waltham, MA, USA; A12379) was used at 1:1000 dilution. Mouse anti-Pericardin antibody (EC11; Developmental Studies Hybridoma Bank, Iowa City, IA, USA) was used at 1:500 dilution followed by Alexa Fluor 647 secondary antibody (Thermo Fisher, Waltham, MA, USA; A21235) at 1:1000 dilution. Confocal images were acquired using a ZEISS LSM900 microscope with a 63X Plan-Apochromat 1.4 N.A. oil objective (ZEISS, Jena, Germany), and ZEN blue edition (version 3.0) acquisition software. Segment A3 of the heart was imaged by collecting Z-stacks. Control groups were imaged first to establish the laser intensity and exposure time for the entire experiment. The exposure time was based on image saturation (at a set point of approximately 70% of maximum saturation) to enable the comparison of fluorescence intensity across all genotypes. ImageJ (version 1.52a) (Schneider et al., 2012) was used for processing. Six adult flies were imaged per genotype, and representative images are shown in the figures.

### Heart structural analysis and quantitation

*Drosophila* heart cardiac myofibril density, cardiomyocyte number, and Pericardin deposition were quantified as previously described (Zhu et al., 2017a). For quantitative comparisons, we analyzed six adult flies (4-day-old females) for each genotype. ImageJ software (version 1.52a) (Schneider et al., 2012) was used to process the images. The Z-stack projections were screened, and image levels containing cardiac myofibrils were selected for analysis while avoiding the ventral muscle layer that underlies the heart tube. The cardiac myofibril number was quantified by using the MyofibrilJ plugin for Fiji (version 1.53q) (Spletter et al., 2018). The entire heart region in segment A2 was selected using the freehand selection function in Fiji to count the number of cardiac myofibrils. For quantitation, cardiac myofibrillar density was calculated as the cardiac myofibril number divided by the size of the heart region. Cardiomyocyte numbers were manually counted in a standard-sized area in heart segment A3. Pericardin deposition was measured in heart segment A3 based on the fluorescence intensity. Cardiac myofibril density, cardiomyocyte number, and Pericardin deposition were each normalized to the values obtained in the control flies.

### Optical coherence tomography (OCT)

Cardiac function in adult *Drosophila* was measured using OCT. The system (Bioptigen) was built as described by the Biophotonics Group, Duke University, NC, USA (Choma et al., 2006; Yelbuz et al., 2002). Adult flies (4-day-old) were anesthetized by carbon dioxide (CO_2_) for 3-5 min and females were preselected from each group. Each fly was gently placed on a plate with petroleum jelly (Vaseline) for immobilization with the dorsal aspect facing the OCT microscopy source and then rested for at least 10 min to ensure the fly was fully awake. For each genotype, ten control and ten RNAi-expressing flies were used. OCT was used to record the adult heart rhythm and heart wall movement in the same position, i.e. the cardiac chamber in the abdominal segment A3 of each fly. Each measurement was obtained in three different positions within the abdominal segment A3, and these were averaged to obtain the cardiac diameter for that fly. M-mode images recorded the heart wall movement during the cardiac cycle. ImageJ software (version 1.52a) (Schneider et al., 2012) was used to process the images. The diastolic dimension and systolic diameter were processed, measured, and determined based on three consecutive heartbeats. The heart period was determined by counting the total number of beats that occurred during a 15-s recording and then dividing 15 by the number of beats.

### Methylation levels

Adult flies (4-day-old females) were dissected and fixed for 10 min in 4% paraformaldehyde in phosphate-buffered saline (1X PBS). Primary antibodies were incubated overnight at 4°C in 2% BSA (Sigma, St. Louis, MO, USA) with 0.1% Triton-X (Sigma, St. Louis, MO, USA) in 1X PBS. Following washes, secondary antibodies were incubated for 2 h at room temperature in 2% BSA (Sigma, St. Louis, MO, USA) with 0.1% Triton-X (Sigma, St. Louis, MO, USA) in 1X PBS. Rabbit anti-H3K4me1 (Abcam, Cambridge, UK; ab8895) and rabbit anti-H3K4me2 (Abcam, Cambridge, UK; ab7766) were each used at 1:2000 dilution, followed by the Alexa Fluor 568 secondary antibody at 1:1000 dilution (Invitrogen, Waltham, MA, USA; A11011). Confocal imaging was performed using a ZEISS LSM900 microscope with a 63X Plan-Apochromat 1.4 N.A. oil objective (ZEISS, Jena, Germany). Images were obtained using ZEN blue edition (version 3.0) acquisition software. Segment A3 of the heart was imaged by collecting Z-stacks. Control groups were imaged first to establish the laser intensity and exposure time for the entire experiment. The exposure time was based on image saturation (at a set point of approximately 70% of maximum saturation) to enable the comparison of fluorescence intensity across all genotypes. ImageJ (version 1.52a) (Schneider et al., 2012) was used for processing.

For quantitative comparison of the nuclear methylation levels, cardiac cells in segment A3 were analyzed. A single image typically captured four cells, equaling four nuclei. We obtained images from six flies for each genotype (RNAi or control), then used the mean fluorescent intensity in the nucleus of ∼20 heart cells total (3-4 per fly) to determine the nuclear methylation level. Finally, nuclear methylation levels were normalized to the value obtained from the control flies. ImageJ (version 1.52a) (Schneider et al., 2012) was used for processing. Representative images are shown in the figures.

### Statistical analysis

Statistical tests were performed using PAST.exe software [Natural History Museum, University of Oslo (UiO), Oslo, Norway]. Data were first tested for normality using the Shapiro-Wilk test (α=0.05). Normally distributed data were analyzed by a one-way analysis of variance (ANOVA) followed by a Tukey-Kramer post-test for comparing multiple groups. Non-normal distributed data were analyzed by a Kruskal–Wallis H-test followed by a Dunn's test for comparisons between multiple groups. Values are presented as mean±standard deviation (s.d.) Statistical significance was defined as *P<0.05. Details for sample sizes used for quantitation have been provided in the figure legends.
